# Current Clinical Laboratory Challenges to Widespread Adoption of Phage Therapy in the United States

**DOI:** 10.3390/antibiotics14060553

**Published:** 2025-05-29

**Authors:** Ahnika Kline, Ana G. Cobián Güemes, Jennifer Yore, Chandrabali Ghose, Daria Van Tyne, Katrine Whiteson, David T. Pride

**Affiliations:** 1 Department of Pathology, University of California, San Diego, CA 92093, USA; 2 Department of Medicine, University of California, San Diego, CA 92093, USA; 3 Center for Innovative Phage Applications and Therapeutics, University of California, San Diego, CA 92093, USA; 4 Bioharmony, Inc., New York, NY 10016, USA; 5 Division of Infectious Diseases, University of Pittsburgh School of Medicine, Pittsburgh, PA 15261, USA; 6 Center for Innovative Antimicrobial Therapy, University of Pittsburgh School of Medicine, Pittsburgh, PA 15261, USA; 7 Department of Biology, University of California, Irvine, CA 92697, USA

**Keywords:** bacteriophage therapy, synergy testing, antibiotic–phage synergy, antibiotic alternatives, clinical microbiology laboratory

## Abstract

The resurgence of phage therapy in Western societies has been in direct response to recent increases in antimicrobial resistance (AMR) that have ravaged many societies. While phage therapy as a concept has been around for over 100 years, it has largely been replaced by antibiotics due to their relative ease of use and their predictability in spectrum of activity. Now that antibiotics have become less reliable due to greater antibiotic resistance and microbiome disruption, phage therapy has once again become a viable and promising alternative, but it is not without its challenges. Much like the development of antibiotics, with deployment of phage therapeutics there will be a simultaneous need for diagnostics in the clinical laboratory. This review provides an overview of current challenges to widespread adoption of phage therapy with a focus on adoption in the clinical diagnostic laboratory. Current barriers include a lack of standard methodology and quality controls for phage susceptibility testing and selection, the absence of phage-antibiotic synergy testing, and the absence of standard methods to assay phage activity on biofilms. Additionally, there are a number of lab-specific administrative and regulatory barriers to widespread phage therapy adoption including the need for pharmacokinetic (PK) and pharmacodynamic (PD) assays, methods to account for changes in phages after passaging, an absence of regulatory guidance on what will be required for agency approvals of phages and how broad that approval will apply, and the increased need for lab personnel or automation to account for the work of testing large phage libraries against bacteria isolates.

## 1. Introduction

What is phage therapy? Phage therapy is the treatment of clinical bacterial infections with bacteria-targeting viruses called bacteriophages. It is a treatment that dates back over 100 years [1,2,3]. Phages were not commonly used to treat bacterial infections until the early 1940s when they were deployed primarily in Eastern Europe and in the Soviet Union for the treatment of bacterial infections [4]. Phage clinical trials even predate the deployment of the first antibiotics [5]: sulfonamides in 1932 [6] and penicillin in 1943 [7]. Critical reviews of phage products [8] and the widespread adoption of antibiotics in the 1950s, caused a shift away from phages [9]. From there, the development of phage therapy lagged behind antibiotics significantly, particularly in the United States, with phages confined to only a few enclaves in Eastern Europe by the late 1900s [10,11].

Only recently has phage therapy seen a rebirth in the United States, largely due to antibiotic resistance reaching a critical mass [12,13]. The number of antibiotics in the pipeline that deploy new mechanisms to kill bacteria are insufficient to address the growing threat of increasing antimicrobial resistance (AMR) [14]. This is significant because many of the antibiotics currently in use in medicine, animal husbandry, and agriculture use similar mechanisms of action [15]; because of the shared mechanisms, resistance to one antibiotic within a class frequently results in resistance to multiple antibiotics within that class [16]. Thus, new therapeutics that can treat infections caused by antibiotic-resistant bacterial pathogens are desperately needed.

## 2. Existing Milestones in Phage Therapy

### 2.1. Phages Are Abundant

It is not difficult to identify phages that are capable of killing many bacterial pathogens. Because phages are naturally occurring, they are often found in the environment. They are also readily available in the human virome, where they likely exist in the trillions [17,18,19]. Just as many bacterial pathogens are found in the human body, their phages are also found in the body. Thus, in searching for phages capable of killing human pathogens, it is useful to examine sites such as sewage, which is often a rich source of phages that target human-associated bacteria [20]. Rich sources of phages typically include sewage, rivers, ponds, lakes, run-off water, soil, animal feces, and human specimens such as body swabs, urine, and saliva [21,22,23,24].

### 2.2. Successful Trials and Experience with Phage Therapy

There exists a significant dichotomy in the wide adoption of phage therapy between Russia and Georgia compared to Europe and the United States. The Eliava Institute in Georgia continued to push phage therapy forward with wide adoption after the interest in phages subsided in the West [25]. Today, pre-prepared cocktails can be purchased in Georgia and Russia without a prescription [26,27]. Outside of these countries, phage therapy remains restricted to compassionate use and clinical trials [28]. However, the collective continued publication of outcomes of patients treated in countries where phages are widely adopted and of clinical trial results supports the clear utility of phage therapy [29,30,31,32]. In the USA, the most advanced ongoing trial is an active phase 2/3 randomized double-blind active-controlled trial for phage therapy for urinary tract infections caused by drug resistant *E. coli* [33]. There are now a handful of phage therapy centers in the USA, matched by those in Europe and Australia (Table 1) [26]. There are also now many companies in the US and Europe emphasizing clinical phage development [34,35].

### 2.3. Promising Results for Phage Therapy for Treating Resistant Bacteria

Now that AMR (Antimicrobial Resistance) has reached a critical mass, physicians commonly observe AMR bacteria in their clinical practice. For example, there are seven pathogens called the ESKAPEE pathogens (*Enterococcus faecium*, *Staphylococcus aureus*, *Klebsiella pneumoniae*, *Acinetobacter baumannii*, *Pseudomonas aeruginosa*, *Enterobacter* spp., and *Escherichia coli*) that are known to commonly harbor AMR and are responsible for the preponderance of nosocomial infections [36,37]. While some of these infections can be treated with commonly used antibiotics, their increasing acquisition of AMR and increasing virulence has necessitated a search for alternative means for treatment [38,39]. There now are a number of institutes across the world that specialize in providing services to individuals who are seeking phages to treat infections caused by these multi-drug resistant (MDR) or extensively drug resistant (XDR) and hypervirulent bacteria [40,41,42,43,44,45]. Many of these institutions interface directly with physicians and hospitals to provide phages for treatment, but some work directly with patients.

In the United States and Western Europe, the concept of phage therapy has seen a rebirth in the past decade [46], largely due to the need impressed upon physicians by the crisis of AMR. While the microbiology observed at most centers likely represents local AMR and bacteria trends, some patterns are consistent across centers. A retrospective review of 100 cases treated with phage therapy throughout Europe demonstrates that most phage therapy interventions targeted patients with *P. aeruginosa* infections, followed by *S. aureus* [47]. At our center in the Western U.S., the majority of requests are for phages targeting *P. aeruginosa*, followed by *S. aureus*, *K. pneumoniae, E. coli*, and *A. baumannii* (Table 2). Not all of these requests result in successful identification of lytic phages and the initiation of treatment, but they highlight the bacterial pathogens that are emerging as target organisms driving the need for phage therapy. Even mycobacteria, which can replicate intracellularly, develop AMR and often require three to four drug regimens over many months of therapy, have shown promise as a target for phage therapy [48,49,50,51].

### 2.4. The Existence of a Variety of Laboratory Methods for Phage Selection

With over one hundred years of experience with phage biology, it is not surprising that reliable laboratory methods for isolating and testing phage have been developed. The existing literature is rich with primary publications and reviews detailing phage selection and susceptibility testing methodologies [52]. Most phage research laboratories employ solid media plaque assays and/or liquid media growth kinetic assays to assess phage activity [53]. Both methods have been successful in identifying phages for clinical use. Whether one chooses to examine susceptibility via solid media or broth testing or both may depend on the personal preference or the available equipment at a particular site [53,54,55]. Additionally, there is wide support for the use of efficiency of plating (EOP) analysis to determine whether a bacterial host is phage-susceptible when performing solid media assays [56]. EOP depends upon using a phage production host where there is high lytic capacity and comparing the efficiency of lysis of the phage on the production host to a bacterial isolate acquired from an infected patient. If the EOP is >0.001 then that phage is considered to have great enough efficiency to be used in the patient for phage therapy [56,57]. While this theory has not been rigorously tested in clinical trials, it represents a current guideline for the use of phages in the treatment of patients [48]. A caveat of using EOP values is that a phage with low titer on both the production host and the target clinical isolate can still produce an EOP > 0.001, but the phage may not be effective therapeutically [58].

## 3. Methodology Challenges to Phage Testing in the Clinical Microbiology Laboratory

### 3.1. Analysis in Liquid Solution Compared to Solid Medium

Some phages may show greater lytic activity on solid media compared to liquid media, and vice versa [59], yet there is currently nothing known about which circumstance might predict greater efficacy in the human host. Most studies and clinical trials take an either/or approach to examining phage lytic potential [60]. In general, when we examine the lytic potential of a phage for a host, we examine not only whether the phage is capable of lysing its host on solid medium by examining EOP, but we also examine its ability to inhibit bacterial growth longitudinally in liquid culture [58]. We took this approach in a recent outbreak of extensively drug-resistant *P. aeruginosa* found in contaminated eyedrops which took four lives and rendered at least 14 people blind [58,61,62]. We were able to identify highly effective jumbo phages, which historically have very little activity in liquid media, through this dual method approach [63]. Jumbo phages have large >200 kp genomes shielded by nucleus-like structures which helps protect them from anti-phage defense mechanisms employed by bacteria, such as restriction–modification systems, and CRISPR-Cas systems, which often leads to broader host ranges [64,65,66,67,68]. While this is an intensive workup of a single isolate for each patient, in the absence of guidelines to support a single assay, the safer choice is to evaluate phage susceptibility against the host via multiple different means to assess activity until consensus methods are established for specific phage/host pairs [69]. Phage plaque assays on solid medium are likely more easily adapted to clinical microbiology laboratory workflows due to the lack of reliance on sophisticated equipment but can be subject to user variability in interpretation of results. They can be adapted to imaging protocols to remove user variability in interpretation of plaque lysis [70], though such a step would incorporate additional expenses and time.

### 3.2. Establishment of Quality Control Bacteria and Phages

The absence of standardization is already problematic for the relatively small number of institutions that currently offer phage therapy, and many have identified that minute differences in protocols can lead to significant differences in phage susceptibilities. Two laboratories using the exact same protocols and host strains will often find that the same phages have different host ranges [59]. There are also differences in protocols regarding whether/when to evaluate if a pathogen has changed its phage susceptibility [71,72]. Standardization of approaches is critical to reduce variability between laboratories, and to achieve consensus in how phage activity is described [55,73]. The variability between laboratories is expected at this moment, as most laboratories often work with different bacterial isolates, different phages, and different testing modalities. Use of different bacterial isolates is difficult, if not impossible to change, as each institution has its own unique patient population with their own pathogens. However, positive and negative quality control bacteria and standard quality control phages to be used in assays can be established across institutions.

### 3.3. The Need for a Standards Committee

Developing a universal standard for phage testing could be accomplished by putting together an advisory board of phage experts with experience in different testing modalities with the goal to develop practical and reproducible testing standards. We applaud that EUCAST (the European Committee on Antimicrobial Susceptibility Testing) recently convened a subcommittee dedicated to developing standards for susceptibility testing of phages with member representatives from 16 different countries [74]. The Antibacterial Resistance Leadership Group (ARLG) also recently published suggested answers to a number of clinical questions related to the use of phage therapy in clinical practice, including what parameters phage susceptibility testing platforms should consider. The group identified similar issues with standardization of methodology, controls, and even storage temperatures of reagents [75]. Consensus methods must ultimately be viable to be reproduced in a clinical microbiology facility, i.e., experiments that require sophisticated and expensive equipment, and high-level scientific training would not be preferred.

### 3.4. The Need for Antibiotic Synergy Testing

It is currently required that patients receive standard-of-care antibiotics during treatment with phage therapy [76]. It is currently unclear how much of a patient’s response is due to phages, antibiotics, or the additive/synergistic effect of combining them together. A number of different studies indicate that synergistic interactions between antibiotics and phages frequently occur [77,78,79,80,81,82,83,84,85], but when it comes to treatment protocols for patients, these concepts appear to be rarely considered. Antibiotics and phages could also act antagonistically. The primary reasons are that: (1) protocols for synergy testing are generally not standardized across laboratories, (2) it is unknown whether in vitro antibiotic–phage synergy correlates with in vivo efficacy, and (3) most clinical laboratories are not properly equipped to perform synergy testing. As is generally the protocol in clinical microbiology laboratories globally, standardization of assays when possible is the key to assay development. Nonetheless, several available assays for phage-antibiotic synergy testing hold promise.

Liquid-based assays have generally been the mainstay for assessing phage-antibiotic synergy [80,86,87]. These assays involve setting up a plate (often a 96-well plate) with a grid of antibiotics and phages at different concentrations along the different axes on the plate, allowing the user to evaluate whether there is greater inhibition of growth of a target pathogen treated with antibiotic and phage together compared to control wells containing either agent alone. This technique has been used to demonstrate synergistic interactions that occur between antibiotics and phages for pathogens such as *Enterococcus* spp. [77,88], *S. aureus* [89], *E. coli* [79], *A. baumannii* [90], *Salmonella* spp. [91], and *P. aeruginosa* [78]. Liquid-based assays have also been used to demonstrate synergy between phages and the anticancer drug, mitomycin C, which highlights their utility in new antimicrobial discovery [92]. While these assays can be useful for demonstrating that antibiotics and phages can work together in vitro and suggest that they may work together in vivo as well, they are cumbersome for clinical laboratories to perform. Most clinical laboratories do not have the necessary equipment, such as plate readers, to perform such assays.

More recently, members of our group have demonstrated that synergistic interactions between antibiotics and phages could be observed in a more simplistic fashion, using a solid media assay rather than more sophisticated liquid-based assays [72]. The benefits of such an assay would be that it would not require expensive equipment to develop, would be relatively easy to interpret, would follow a straightforward model for predicting synergy, additivity, antagonism, and indifference, and would fit within the bounds of what could easily be developed in clinical microbiology labs. Using this protocol, our group found that trends in synergy could be established for both Gram-positive and Gram-negative pathogens and confirmed that pre-existing antibiotic susceptibility was not required for synergy to be observed. Specifically, we confirmed the previous observation made using liquid cultures that vancomycin-resistant *Enterococcus* became susceptible when combined with phage [77,88,93]. The drawback of this assay is that it requires phage strips and antibiotic strips to be constructed by a manufacturer to ensure quality and reproducibility. We recommend that pathogens be evaluated for antibiotic-phage synergy, even in cases where resistance to those antibiotics exists in the source pathogen, as this will provide more information to the treating physician.

## 4. Phage Therapy Treatment Differences That May Influence Laboratory Methodology

### 4.1. Cocktails vs. Individual Phages

There are many strategies to deliver phage therapy to individuals, but two have emerged as the primary strategies employed by most treatment centers. In the first strategy, multiple different phages often targeting different receptors are combined into a cocktail and employed simultaneously to attack the same bacterial pathogen. The rationale behind this strategy is to create a higher barrier to mutation in any single bacteria that would render it resistant to all of the cocktail phages at once and may also better account for heterogeneity in even clonal bacterial populations [94,95,96,97]. As quickly as phages for human bacterial pathogens can be found, bacterial resistance to those phages can develop, and may explain why phage treatment protocols often employ cocktail therapies [5,98]. In the second strategy, an individual phage is employed against a bacterial pathogen and is then replaced by another individual phage only if the pathogen develops resistance to the first phage in a staged approach, with the advantage of potentially eradicating the infection with fewer phages and reduced cost [99,100]. The use of a single phage may decrease the chance of an adverse host immune response compared to the use of a phage cocktail [101]. In each of these scenarios, laboratory testing is necessary to identify whether the bacterial pathogen in question is susceptible to the phages being used. Additional information is also needed, including the mechanism of action and/or the receptors that each phage employs to infect the bacterial host, to ensure that resistance to one phage does not convey resistance to multiple phages within the cocktail or resistance to phages that may be used subsequently in staged approaches. Unfortunately, both approaches can require significant labor for a clinical laboratory to determine susceptibilities for multiple different phages among single or multiple bacterial pathogen hosts.

Phages use a number of bacterial virulence factors as receptors [102]. However, identification of the receptors for phages prior to using them creates a significant burden for most laboratories given the expense and labor involved in developing phages for clinical use. Particularly when using phage cocktails where each phage may employ a different receptor, each receptor should be identified separately prior to use. Even genetically unrelated phages might employ the same receptors [103,104], and thus, even when employing a technique such as a staged approach for phage therapy, receptors likely need to be identified beforehand to reduce the potential for cross-resistance [105].

One prominent issue for evaluating phage therapy for both cocktail and staged approaches is the potential for mechanisms that reduce likelihood of lysis across multiple phage types. For example, a prior study characterizing resistance to a group of *Enterococcus* phages found that phage resistance developed via membrane mechanisms that generally made the bacteria less accessible to the phages [106]. While these mechanisms were not likely to represent the actual receptors for the phages, the lack of access to their receptors resulted in cross-resistance to a number of different types of phages. When evaluating the development of phage resistance, it is important to note whether mutations might occur that result in resistance to a variety of phages through mutations in similar genes in the same biosynthetic pathways [107,108].

### 4.2. Phage Therapy for Multispecies Infections

Phages are thought to be attractive for single species infections because their specificity may limit killing of normal microbial flora [109]. Because many infections, such as intraabdominal infections, are caused by multiple bacteria simultaneously, phage therapy approaches that target multiple bacteria simultaneously have been investigated. These approaches generally combine multiple phages targeting different bacteria into a single cocktail [110,111,112,113,114]. There also exist some polyvalent phages, which target multiple bacteria species or genera [115,116]. For example, polyvalent phages have been discovered that target multiple diarrheal pathogens simultaneously [117], and some that target multiple Salmonella serovars [118,119]. Recent improvements in sequential multi-host isolation methods make the selection of polyvalent phages more feasible [120,121]. The presumption is that a polyvalent phage would be tested in vitro against each of the desired multiple bacterial targets from a polymicrobial infection individually prior to its selection for use. The clinical laboratory would then test each host pathogen for susceptibility to the polyvalent phage prior to use in phage therapy. Phages with broad host ranges may lose a key advantage of phage therapy over antibiotics in sparing the host bacterial microbiome, so polyvalent phages also need to be evaluated against known commensal organisms, to ensure they do not increase adverse outcomes caused by disruption of normal flora, such as C. difficile colitis [122].

### 4.3. Assays for Phages Used to Treat Biofilms

Biofilms remain largely impenetrable to standard antimicrobial therapy. In clinical practice, the mainstay of treatment for infections predominated by biofilms like catheter-associated infections or prosthetic hardware infections remains removal of the material containing the biofilm [123]. Numerous in vitro and ex vivo models have shown biofilm disruption by phages [124]. A number of clinical cases have shown promise with the addition of phages to existing device removal strategies in prosthetic joint infections [125]. Nonetheless, strategies to harness phages to disrupt biofilms are under investigation [126]. No FDA approved diagnostics exist to assay biofilm degradation by phages. Most researchers assay biofilm lysis by measuring crystal violet uptake in 96 well plates after phage exposures [127,128], which could be adapted to the clinical lab, but generally requires equipment that is not always present. Automated confocal microscopy images hold promise in research labs, but are unlikely to be developed into clinical assays [129,130]. As many others have highlighted, the development of an alternative assay for biofilm measurement that does not require expensive equipment, is necessary for biofilm assays to become routine measurements to characterize phage activity in the clinical laboratory [131].

### 4.4. Accounting for Phage Passaging

Because phages are biological entities, propagation of the “same” phage across different laboratories can lead to the accumulation of mutations and divergence, which has implications for phage activity. Optimal phage amplification methods also vary depending on the phage [132]. Clinical laboratories need to be able to assay for lot-to-lot variability caused by phage passaging as part of phage optimization. For example, to produce phages with broadened host ranges, laboratories may perform phage training experiments [120,133]. The Appelman’s protocol involves passaging phages iteratively on bacteria isolates, including resistant ones, until the phage evolves with an improved ability to lyse the bacterial host [134]. Many of the phages used in the 100-phage therapy case study set in Belgium [47] used a modified Appelman’s protocol [135]. Another method used in vitro involves co-evolution of phage and host over successive generations, where both the host and the phage evolve together. While the method can result in extinction events for either the host or the phage, it can also result in a stalemate between host and phage where neither is capable of eradicating the other. The important part of this method is that when the trained phage is taken from this experiment and is exposed to the untrained host, the untrained host is often incapable of adapting to the trained phage [136]. By accelerating evolution between host and phage without recombinant manipulation, researchers were able to generate phages with superior abilities to eradicate untrained *E. coli* hosts and with expanded phage host ranges. Phage optimization experiments could become a standard part of phage development, particularly as researchers seek to extend host ranges of phages to mirror those of antibiotics. These tools show how rapidly phages can evolve through successive passaging, and highlight the need for assays in the clinical laboratory to verify that prepared phages still perform as expected prior to their clinical use, including evaluating standard quality control phages for consistency. This type of rapid evolution does not exist with antibiotics, where laboratories and clinicians need not usually worry about batch-to-batch variation.

### 4.5. The Need for Lytic Phages

Though lysogenic phages may hold future promise [137], therapeutic phages used currently are strictly lytic, which means they do not have the capacity of integrating into the bacteria genome. Genome sequencing and annotation are needed to confirm that lysogenic genes or pathogenicity factors are not present in candidate phage genomes, and that the phages are appropriate for therapeutic use [138,139]. This sequencing need creates a barrier to the creation of new phage libraries outside of a handful of centers, but we feel that pre-developed phage libraries could still be tested for susceptibility within clinical labs.

## 5. Organization and Regulatory Barriers to Widespread Phage Testing in Clinical Microbiology Laboratories

### 5.1. Automation and Personnel Issues

A trend that is occurring throughout the world is the development of total laboratory automation (TLA) [140]. Utilization of automation protocols, particularly in the processing of traditional cultures, has helped to reduce the hands-on time necessary for the processing of cultures [141], accelerate the time to results [142], and allows hospital systems to adjust to aging work forces that are difficult to replace [143]. Automation has helped to reduce manual tasks in many labs across the world, which is in stark contrast to the development of phage testing in the clinical laboratory. To adapt the clinical laboratory to phage testing, the laboratory would either need dedicated research staff to perform this type of testing or have the ability to automate phage testing. Right now, there are no known clinical laboratories that have automated phage susceptibility testing. The approved assays for routine antibiotic susceptibility that have been developed for total lab automated platforms are based on disk diffusion assays on solid medium [144]. While such assays could theoretically be developed for phages to measure zones of inhibition, no universal standards have been developed. Broth microdilution modules for TLA are in the development pipeline, but will be only compatible with susceptibility plates developed by the parent company [145]. Therefore, it is incumbent upon regulatory bodies such as EUCAST and CLSI (the Clinical Lab Sciences Institute) to begin to develop standardized processes that can then be automated with results available to clinicians in the same timeframe as standard antibiotic testing (24 h), and available to repeat if the patient continues to grow the bacteria despite therapy.

### 5.2. Concurrent Development of PK/PD and Anti-Phage Antibody Assays

In any clinical drug development, Pharmacokinetic and Pharmacodynamic (PK/PD) assays are part of dose finding studies. In many antimicrobials therapy regimens, such as aminoglycosides, vancomycin, and triazoles, measurement of blood levels is a standard of therapy [146,147,148], and usually performed in a chemistry laboratory. Phages are self-replicating with complex PK/PD profiles [149,150]. As such standardizing the methods for assessing phage concentrations in blood is also an area of research, with many labs preferring qPCR methodology [151]. Given the existing qPCR assays are often performed in the clinical microbiology laboratory, it should be expected that the laboratory may need to simultaneously bring on phage qPCR assays to help determine individualized phage dosing, and will require collaboration with clinical chemists and pharmacists. At the same time, phage specific immunity may hinder treatment efficacy and clinical assays to look for phage neutralizing antibodies are likely to be necessary.

### 5.3. Regulatory Hurdles

**Figure 1:**
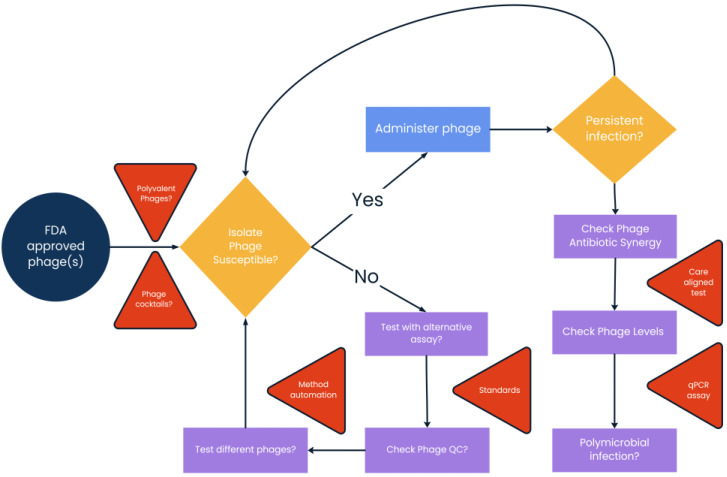
A proposed schematic for phage susceptibility testing in the clinical laboratory. Phage products will be tested against patient isolates for activity. Methods may depend on the phage product makeup. Phage resistance should lead to secondary testing with additional standardized assays, QC assurance that the phage has not mutated through passaging, and/or selection of new phage(s) for testing. Automated methods are necessary to assure sufficiently rapid test result times. Breakthrough infection will require repeat testing to assure no development of isolate resistance, synergy testing aligned with the antibiotics used in the patient’s care, measurement of phage serum levels (through qPCR assays) and confirmation that there are not additional organisms contributing to the infection. Red triangles highlight current needs and challenges to this proposed clinical testing workflow.

At present, phages are only approved in the United States for pesticide applications (by the EPA), for food applications (by the FDA and USDA) and are used in FDA-approved phage based diagnostic assays [152]. Phages are not FDA approved for human use in the USA and are only available through clinical trials and through expanded access programs [153]. Despite the development of polyvalent phages, phage cocktails, and phage-antibiotic synergy approaches, all of which may protect against the development of phage resistance and allow for phage development centers to consolidate applications to the FDA, we believe that the eventual breadth of available phages for treatment will surpass the number of antibiotics available. The best current correlate to this individuality and simultaneous breadth of product comes from gene therapy treatments such as CAR-T cell therapies, in which regulatory agencies determine how much clinical and pre-clinical data are required to approve each successive treatment [154,155]. We envision that individual phages may be regulated for clinical use in a similar fashion, and support that the FDA has entertained alternative trial designs for phages [156]. The need for high throughput assays to help providers select the correct individual phages will be paramount. If the sheer breadth of phages that have already been used in the current models of phage therapy come to market, it may make the eventual regulatory approval of widely deployable susceptibility testing assays for phages quite costly. Even if susceptibility testing is able to be implemented as a lab-developed test (LDT), the work to validate LDTs presents a barrier to widespread deployment. This increases the likelihood that phage selection, testing and therapy remains restricted to a few individual centers. As a start, recognizing the novelty of phage therapy and moving towards a different model of regulatory approval for phage susceptibility testing would allow for a wider network of phage susceptibility testing and better access to phage therapy for patients (Figure 1) [157].

## 6. Conclusions

Phage therapy holds significant promise for changing the landscape of how infections caused by bacterial pathogens are treated. Developing virulence and AMR in the community and in hospitals is widely driven by the use and availability of antibiotics [158]. A substantial decrease in the use of antibiotics resulting from the use of phages as an alternative could cause a dramatic shift in the current growth projections of AMR. To achieve this, physicians must be convinced to change their antimicrobial prescription practices and shift towards more widespread use of phages. Currently, however, the phage field has not yet proven the utility of phage therapy in the USA through large, randomized controlled trials. Additionally, phage host ranges generally do not rival antibiotics, though the disruption to normal microbial flora may be less as a result [159]. Production of phages is challenging at most facilities [160]. Furthermore, general skepticism remains about the ability of phages to cure infections. Phage research lacks investment as a field as compared to other pharmaceuticals, but is gaining ground in the antibiotic space [161]. Finally, the general public is only now beginning to appreciate the looming AMR crisis [162]. Once these issues are addressed, the more practical challenges to implement phage testing in the clinical laboratory, as described in this manuscript, will be front and center.

## Figures and Tables

**Table 1:** Phage centers.

Center	Associated University or Hospital	Location	Website
Center for Innovative Phage Therapeutics and Applications (IPATH)	University of California, San Diego	San Diego, CA, USA	https://sites.medschool.ucsd.edu/som/medicine/divisions/idgph/research/center-innovative-phage-applications-and-therapeutics/Pages/default.aspx (accessed on 5 May 2025)
Center for Phage Technology (CPT)	Texas A&M University	College Station, TX, USA	https://cpt.tamu.edu/history-and-mandate/ (accessed on 5 May 2025)
TAILOR Labs	Baylor College of Medicine	Houston, TX, USA	https://www.bcm.edu/research/research-centers/tailor (accessed on 5 May 2025)
Center for Phage Research and Therapy at Yale	Yale University	New Haven, CT, USA	https://phage.yale.edu/ (accessed on 5 May 2025)
Pittsburgh Phage Program	Children’s Hospital of Pittsburgh	Pittsburgh, PA, USA	https://www.i4kids.org/research/pittsburgh-phage-progam-p3 (accessed on 5 May 2025)
Eliava Phage Therapy Center	Eliava Institute of Bacteriophages, Microbiology, and Virology	Tbilisi, Georgia	https://eptc.ge/ (accessed on 5 May 2025)
Phage Therapy Unit of the Medical Centre of the Institute of Immunology and Experimental Therapy PAS	Polish Academy of Sciences	Wroclaw, Poland	https://hirszfeld.pl/en/structure/iitd-pan-medical-center/phage-therapy-unit/ (accessed on 5 May 2025)
Israeli Phage Therapy Center of Hadassah	Hebrew University of Jerusalem and Ein Kerem Hospital	Jerusalem, Israel	https://phageil.com/the-israeli-phage-therapy-center/ (accessed on 5 May 2025)
Phage Therapy Institute at Waseda University	Waseda University	Tokyo, Japan	https://www.waseda.jp/inst/cro/en/institutes-list/phage-therapy-institute/ (accessed on 5 May 2025)
Phage Center at Queen Astrid Military Hospital	Queen Astrid military hospital	Brussels, Belgium	https://www.hopitalmilitaire.be/home_fr.php (accessed on 5 May 2025)
Monash Phage Foundry	Monash University	Melbourne, Australia	https://www.monash.edu/impact-amr/phage-therapy (accessed on 5 May 2025)
Shanghai Institute of Phage	Shanghai Public Health Clinical Center of Fudan University	Shanghai, China	
Phage Australia	The Westmead Institute for Medical Research	Westmead, New South Wales	https://www.phageaustralia.org/ (accessed on 5 May 2025)

**Table 2:** Top phage therapy requests for pathogens at IPATH UCSD.

Bacterium	Number of Requests	Number of Phages Found	Number of Cases Treated
*Pseudomonas aeruginosa*	348	67	14
*Staphylococcus aureus*	175	7	4
*Escherichia coli*	131	17	5
*Klebsiella pneumoniae*	125	15	3
*Acinetobacter baumannii*	72	12	12

## Data Availability

No new data were created or analyzed in this study.
